# Supplementary material for: The genome sequence of the larch ladybird beetle, Aphidecta obliterata (Linnaeus, 1758) (Coleoptera: Coccinellidae)
Source: Wellcome Open Res. 2025 Nov 20;10:646. [Version 1] doi: 10.12688/wellcomeopenres.25090.1 (PMC12820478; doi:10.12688/wellcomeopenres.25090.1)
Supplement: Peer review history

# Reviewer report: Reviewer response for version 1

**DOI:** 10.21956/wellcomeopenres.27660.r145104

**Published:** 2026-01-22

**Authors:** Christophe Klopp

**Affiliations:** 1 INRAE, Castanet-Tolosan, France

The authors present a chromosome-level genome assembly for *Aphidecta obliterata*, or the larch ladybird. The sequencing and assembling methods are sound.

 Using the primary option when Hi-C is available is outdated. The authors could have improve their assembly haplotyping by running hifiasm with the Hi-C read files. 

 They could also have produced a second haplotype scaffolded in chromosomes using ragtag and the first haplotype assembly as matrice. This is common practice nowadays and eases the assembly use.

 Kmer multiplicity graph shows the hap1 (which by the way should be called primary) is missing quite a lot of kmers shared between haplotypes. This is problematic and should be looked into.

 This does not fit with the merqury completeness statistics found in the article. The should also be checked, didn't you swap primary and accessory. 

Are sufficient details of methods and materials provided to allow replication by others?

Yes

Is the rationale for creating the dataset(s) clearly described?

Yes

Are the datasets clearly presented in a useable and accessible format?

Yes

Are the protocols appropriate and is the work technically sound?

Yes

Reviewer Expertise:

Genome assembly and annotation

I confirm that I have read this submission and believe that I have an appropriate level of expertise to confirm that it is of an acceptable scientific standard, however I have significant reservations, as outlined above.

# Reviewer report: Reviewer response for version 1

**DOI:** 10.21956/wellcomeopenres.27660.r141138

**Published:** 2026-01-20

**Authors:** Arun Arumugaperumal

**Affiliations:** 1 Department of Biotechnology, Rajalakshmi Engineering College, Rajalakshmi Engineering College, Thandalam, Chennai 602105, Tamil Nadu, India

The data note describes the genome sequencing of the larch ladybird beetle *Aphidecta obliterata*. The photograph of the beetle has been nicely recorded as figure 1. A male has been sequenced and the sex system observed was XO. Hence, they have reported 9 autosomes and an X chromosome for this male specimen. The assembly size reported is 349.56 Mb. 18,868 protein-coding genes have been annotated through Ensembl. The mitogenome of size 17.95 kb has also been assembled. The authors have used PacBio long-read sequencing and also Hi-C sequencing data to obtain a high-quality genome assembly. The quality is evident from the high N50 values of 4.46 Mb in the case of contigs and 38.8 Mb in the case of scaffolds. The assembly has achieved a BUSCO completeness of 97.6% with respect to the endopterygota_odb10 dataset. The beetle, *Aphidecta obliterata, *feeds on aphids present on pine trees and hence it is a suitable pest controller. The report of the genome sequence of this important beetle adds value to future biological studies. The article can be indexed.

Are sufficient details of methods and materials provided to allow replication by others?

Yes

Is the rationale for creating the dataset(s) clearly described?

Yes

Are the datasets clearly presented in a useable and accessible format?

Yes

Are the protocols appropriate and is the work technically sound?

Yes

Reviewer Expertise:

Bioinformatics; Genomics

I confirm that I have read this submission and believe that I have an appropriate level of expertise to confirm that it is of an acceptable scientific standard.

# Reviewer report: Reviewer response for version 1

**DOI:** 10.21956/wellcomeopenres.27660.r141143

**Published:** 2026-01-06

**Authors:** Panagiotis Ioannidis

**Affiliations:** 1 Foundation for Research & Technology - Hellas, Crete, Greece

This paper describes the genome assembly of the beetle Aphidecta obliterata.

 Generally, it is a very good assembly by all metrics presented; complete BUSCOs > 97%, QV = 61, k-mer completeness > 99%.

 The CC ratio (scaffold number / chromosome number = 200/10 = 20) is a bit large. This is also apparent in the blob plot, where there is a cluster of small scaffolds with varying sequencing coverages and a GC content of ~41% (which differs significantly from the GC content of the large scaffolds at ~36%). This finding definitely needs a comment from the authors; what are these contigs similar to? Is it possible that they're contaminants from bacteria, for example? Are they (very!) repetitive regions that couldn't be assembled?

 To my delight, I also saw that a genome annotation report is included! This is a very good thing because it increases the usefulness of this genome assembly. I believe that all genome assemblies should be annotated these days. However, there is one thing missing; quality report on the predicted gene set! It is absolutely essential to run BUSCO on every predicted gene set. Otherwise, there's no way of determining how good (or bad) a gene set is. For example, it could be missing many essential genes if no suitable transcript and protein evidence was used.

Are sufficient details of methods and materials provided to allow replication by others?

Yes

Is the rationale for creating the dataset(s) clearly described?

Yes

Are the datasets clearly presented in a useable and accessible format?

Yes

Are the protocols appropriate and is the work technically sound?

Yes

Reviewer Expertise:

insect genomics; bioinformatics

I confirm that I have read this submission and believe that I have an appropriate level of expertise to confirm that it is of an acceptable scientific standard.
